# A health resort-based intervention reduces insomnia with minimal changes in quality of life in COVID-19 survivors and non-COVID-19 controls

**DOI:** 10.3389/fpubh.2026.1803380

**Published:** 2026-04-10

**Authors:** Grzegorz Onik

**Affiliations:** Department of Physical Medicine, Chair of Physiotherapy, Faculty of Health Sciences in Katowice, Medical University of Silesia in Katowice, Katowice, Poland

**Keywords:** insomnia, post-COVID-19, quality of life, sanatorium treatment, treatment effectiveness

## Abstract

**Background:**

Persistent post-COVID-19 symptoms, especially insomnia, are common and markedly impair quality of life. While some patients improve over time, many experience long-lasting complaints. Health resort treatment has shown potential benefits in long COVID, but its effects on sleep and quality of life remain unclear. This study aimed to evaluate the impact of comprehensive health resort treatment on insomnia and quality of life in post-COVID-19 individuals and to compare outcomes with those without prior SARS-CoV-2 infection.

**Methods:**

A total of 101 participants, including 30 post-COVID-19 individuals, underwent comprehensive health resort treatment. Propensity score matching was used to compare post-COVID-19 and non-COVID groups. Insomnia and health-related quality of life were assessed using the Athens Insomnia Scale and EQ-5D-5L, respectively, before and after the sanatorium stay.

**Results:**

The Athens Insomnia Score significantly decreased in post-COVID-19 individuals following sanatorium treatment (6.24 ± 5.93 vs. 3.97 ± 4.19 points; *p* = 0.0005). The EQ index did not change significantly after treatment (*p* = 0.08). Overall health status, assessed using the visual analog scale (VAS), significantly improved in individuals with a history of COVID-19 (76.03 ± 12.70 vs. 88.45 ± 7.80 points; *p* < 0.0001). Treatment effectiveness did not differ significantly between the patient groups.

**Conclusions:**

Comprehensive health resort treatments may improve insomnia and self-reported health in COVID-19 survivors. Effects on overall quality of life appear limited, and outcomes are similar to those in individuals without prior COVID-19. Further research is needed to clarify the clinical utility of this approach.

## 1 Introduction

Persistent post-COVID-19 symptoms have been widely documented among recovered individuals, and include cardiovascular, respiratory, gastrointestinal, urogenital, and neurological complications, as well as cognitive impairment and reduced functional capacity (1–4). Neuropsychiatric sequelae are particularly prevalent, with insomnia being the most frequently reported condition, with prevalence estimates ranging from 40 to 76.1% depending on the study (2, 5, 6). Furthermore, Ryu et al. (7) report persistent sleep disturbances in patients with long COVID even 3 years after SARS-CoV-2 infection. Given that sleep disorders may lead to multidimensional consequences, including physical, psychological, and functional impairments (8), Hoang et al. (6) highlighted the need for comprehensive interventions to mitigate insomnia in COVID-19 survivors. Accumulating evidence indicates that these long-term outcomes substantially impair overall quality of life (9–11). Malik et al. (12) reported that 58% of post-COVID-19 patients experience reduced quality of life. Artemiadis et al. (13) further suggested that diminished quality of life in individuals with long COVID may be associated with fatigue and psychological distress. Moreover, factors such as the ability to perform activities of daily living and maintain independence have also been identified as important determinants of quality of life in this population (14). Most available studies assess quality of life in this group over relatively short follow-up periods (12), whereas Szewczyk et al. (15) reported reduced quality of life at a median of 2 years after infection with SARS-CoV-2.

The pathophysiological mechanisms underlying persistent COVID-19 symptoms are multifactorial and remain incompletely understood. Current hypotheses implicate immune system dysregulation, autoimmune processes, microvascular and hemodynamic alterations, as well as disruptions in neural signaling pathways (1). While longitudinal data suggest a gradual reduction in symptom intensity over time in some individuals (16, 17), a considerable proportion of patients continue to experience persistent health issues. In response to the growing burden of post-COVID conditions, multiple therapeutic strategies have been explored, including pharmacological management (1, 18), structured physical activity programs (19, 20), physical and rehabilitative therapies (21, 22), psychological support interventions (23), nutritional supplementation (24), and complementary medicine approaches (25). Given the interplay of these mechanisms, a comprehensive and multifaceted approach is required to effectively manage and alleviate persistent symptoms.

Health resort treatment is a comprehensive therapeutic modality that integrates balneological interventions, physical medicine techniques, massage and structured therapeutic exercise (26, 27). This intervention is delivered in officially designated health resort areas that comply with stringent regulatory standards. Its clinical relevance is determined by the therapeutic properties of the local climate and the availability of natural therapeutic resources, including mineral waters and peloids, regardless of the geographical location of the sanatorium (e.g., lowland, coastal, or mountainous settings) (26–29). Beyond its direct clinical effects, health resort treatment may also be associated with additional benefits related to patient participation in structured recreational activities and exposure to social and cultural environments during the treatment period (27).

To date, few studies have examined the impact of balneotherapy on quality of life and sleep quality in individuals with a history of COVID-19 (30–32). Most available studies have assessed only the immediate effects of sanatorium-based treatment, whereas those conducted by Ovejero et al. (30) and Rapoliene et al. (32) also included follow-up assessments. Nevertheless, none of the existing studies included a control group of individuals without SARS-CoV-2 infection who underwent comprehensive sanatorium-based treatment in the same setting. Therefore, the present study provides an opportunity not only to evaluate the effects of such treatment in post-COVID-19 individuals but also to compare its effectiveness with that observed in individuals without a history of COVID-19. This may contribute to the development of future interventional studies. Accordingly, the present study pursued two objectives. The primary objective was to evaluate the effects of comprehensive health resort treatment on quality of life and insomnia in individuals with a history of COVID-19. The secondary objective was to compare the effectiveness of comprehensive health resort treatment between individuals with and without a history of SARS-CoV-2 infection.

The paper is structured as follows. The first section provides a review of the relevant literature on quality of life and sleep quality in post-COVID-19 individuals, forming the theoretical background and justifying the design and conduct of the study. The second section presents the research methodology, including a detailed description of the applied methods and research tools. The subsequent section is devoted to the presentation and interpretation of the results, as well as a discussion of the study's limitations. The final section outlines the main conclusions drawn from the research.

## 2 Material and methods

### 2.1 Participants

The study included 101 individuals (80 women and 21 men) aged 40–83 years (mean age: 69.42 ± 9.35 years) who underwent comprehensive health resort treatment in 2025. Of these participants, 30 reported a history of COVID-19 infection. Participants were divided into two groups based on COVID-19 history: Group 1 (history of COVID-19) and Group 2 (no history of COVID-19, control group). Detailed characteristics of the participants are presented in Table 1.

**Table 1:** Baseline characteristics of individuals undergoing health resort treatment before propensity score matching, stratified by history of COVID-19.

Anthropometric and clinical variables	All (*n* = 101)			Group 1 (*n* = 30)			Group 2 (*n* = 71)			*t*	*p-*Value
	**Min**	**Max**	**Mean** ±**SD**	**Min**	**Max**	**Mean** ±**SD**	**Min**	**Max**	**Mean** ±**SD**		
Age (years)	40	83	69.42 ± 9.35	40	79	68.97 ± 10.32	43	83	69.61 ± 8.98	0.31	0.96
Height (m)	1.46	1.88	1.65 ± 0.08	1.46	1.83	1.64 ± 0.10	1.53	1.88	1.66 ± 0.09	0.96	0.34
Weight (kg)	54	115	78.36 ± 14.27	59	112	78.93 ± 14.89	54	115	78.11 ± 14.10	−0.27	0.79
BMI (kg/m^2^)	21.63	42.86	28.72 ± 4.27	21.63	40.16	29.35 ± 4.21	21.83	42.86	28.45 ± 4.30	−0.96	0.34
SBP (mmHg)	112	156	141.36 ± 7.27	126	153	140.87 ± 7.51	112	156	141.56 ± 7.21	0.43	0.66
DBP (mmHg)	60	90	77.58 ± 5.78	67	90	79.47 ± 5.14	60	88	76.79 ± 5.90	−2.16	0.04
HR (bpm)	46	98	73.83 ± 8.80	46	88	70.43 ± 10.50	56	98	75.27 ± 7.62	2.59	0.01

Data are presented as mean ± standard deviation (SD) and range (min–max). Differences between groups were assessed using the independent samples Student's t-test.

t, t statistic; p, corresponding p-value.

Despite the absence of significant differences in age, height, body weight, and body mass index before matching, unequal group sizes may have compromised comparability and increased the risk of confounding bias; therefore, propensity score matching was performed to improve baseline balance. The final matched sample comprised 58 individuals (46 women and 12 men) aged 40–79 years (mean age: 68.74 ± 9.36 years) with a mean body mass index of 28.92 ± 3.68 kg/m^2^. In both groups, men constituted 21% of the participants. The mean time since infection among participants with a history of COVID-19 was 3.67 ± 1.04 years. Group characteristics after propensity score matching are presented in Table 2.

**Table 2:** Baseline characteristics of individuals undergoing health resort treatment after propensity score matching, stratified by history of COVID-19.

Anthropometric and clinical variables	Group 1 (*n* = 9)			Group 2 (*n* = 29)			*t*	*p-*Value
	**Min**	**Max**	**Mean** ±**SD**	**Min**	**Max**	**Mean** ±**SD**		
Age (years)	40	79	68.62 ± 10.32	46	79	68.86 ± 8.48	0.10	0.92
Height (m)	1.46	1.83	1.64 ± 0.10	1.53	1.86	1.65 ± 0.08	0.56	0.58
Weight (kg)	59	112	78.41 ± 14.87	60	110	79.14 ± 12.77	0.20	0.84
BMI (kg/m^2^)	21.63	35.44	28.98 ± 3.74	21.97	37.25	28.87 ± 3.67	−0.10	0.92
SBP (mmHg)	126	153	140.48 ± 7.34	126	155	141.66 ± 6.21	0.66	0.51
DBP (mmHg)	67	90	79.52 ± 5.22	62	26	76.55 ± 5.83	−2.04	0.05
HR (bpm)	46	88	70.62 ± 10.63	56	88	75.69 ± 6.82	2.16	0.03

Data are presented as mean ± standard deviation (SD) and range (min–max). Differences between groups were assessed using the independent samples Student's t-test.

t, t statistic; p, corresponding p-value.

Among the individuals included in the analysis, the most frequently observed comorbidity was hypertension, present in 45% of participants. Other common comorbidities included hypothyroidism and varicose veins. No statistically significant differences were observed in the distribution of comorbidities between the groups. The distribution of comorbidities in the groups undergoing health resort treatment is presented in Table 3.

**Table 3:** Prevalence of comorbidities among participants undergoing health resort treatment.

Comorbidity	% of individuals		χ^2^	*p-*Value	Comorbidity	% of individuals		χ^2^	*p*-Value
	**Group 1**	**Group 2**				**Group 1**	**Group 2**		
Osteoporosis	7%	10%	0.22	0.64	GERD	3%	3%	0.00	1.00
Hypothyroidism	34%	14%	3.39	0.06	Hyperuricemia	10%	3%	1.07	0.30
Hypertension	38%	52%	1.11	0.29	BPH	7%	0%	2.07	0.15
Depression	3%	7%	0.35	0.55	Psoriatic arthritis	3%	0%	1.07	0.31
Mitral regurgitation	0%	3%	1.02	0.31	Chronic kidney disease	0%	3%	1.02	0.31
Ischemic heart disease	10%	3%	1.07	0.30	Rheumatoid arthritis	3%	0%	1.02	0.31
Varicose veins	10%	24%	1.93	0.16	Asthma	3%	3%	0.00	1.00
Diabetes mellitus	7%	10%	0.22	0.64	Hyperthyroidism	3%	3%	0.00	1.00
Gout	0%	7%	2.07	0.15	Spondyloarthritis	3%	0%	1.02	0.31

Data are presented as percentages. Differences between groups were assessed using the chi-square test of independence.

χ^2^, chi-square statistic; p, p-value.

### 2.2 Eligibility criteria

Participants who completed a 21-day comprehensive health resort treatment were eligible for inclusion in the analysis. Exclusion criteria included cardiovascular conditions (NYHA class > II heart failure, atrial fibrillation, history of myocardial infarction, coronary artery bypass grafting, percutaneous coronary intervention with stent placement, or pacemaker implantation); neurological disorders (Parkinson's disease, multiple sclerosis, paresis or spasticity, history of stroke, meningitis, or intellectual disability); respiratory diseases (chronic obstructive pulmonary disease, pneumoconiosis); amputations; and cancer.

### 2.3 Treatment regimen

The sanatorium program lasted 21 days. Each patient's treatment schedule was individually tailored based on reported symptoms and findings from the clinical examination at admission. Among the various therapeutic modalities, peloid therapy (mud applications) was the most frequently administered, applied at a temperature of 40 °C for 15 min in 95% of participants. Brine therapy was the second most commonly applied modality, administered at a temperature of 38–40 °C for 15 min to 71% of patients. LED therapy was less frequently employed, applied to only 2% of subjects. No statistically significant differences in the frequency of modality administration were observed between groups. A comprehensive description of the physical medicine and balneological interventions is presented in Table 4.

**Table 4:** Frequency of physical medicine and balneological interventions in patient groups.

Intervention	Group 1 (*n* = 29)	Group 2 (*n* = 29)	χ^2^	*p-*Value
Vacuum massage	62%	62%	0.00	1.00
Classical massage	17%	14%	0.13	0.72
Mechanical massage	14%	21%	0.48	0.49
Pearl bath	17%	14%	0.13	0.72
Peloids	89%	100%	3.16	0.08
Brine	62%	79%	2.08	0.15
Local cryotherapy	17%	14%	0.13	0.72
Whirlpool bath	31%	24%	0.35	0.56
Infrared light	7%	10%	0.22	0.64
Magnetledtherapy	3%	3%	0.00	1.00
TENS	7%	17%	1.46	0.23
Low-level laser therapy	10%	21%	1.18	0.28
Ultrasounds	0%	7%	2.07	0.15
Iontophoresis	10%	7%	0.22	0.64
LED therapy	3%	0%	1.02	0.31

Data are presented as percentages. Differences between groups were assessed using the chi-square test of independence.

χ^2^, chi-square statistic; p, p-value.

Physical medicine and balneological interventions were combined with supervised exercises to improve range of motion, endurance, muscle strength, coordination, and balance. Additionally, participants received health education on maintaining a balanced diet, regular physical activity, and the risks of tobacco, alcohol, and other substance use.

### 2.4 Methods of assessment

Insomnia and quality of life were assessed at two time points: before and after completion of the health resort treatment, during routine medical examinations. The severity of insomnia was measured using the Athens Insomnia Scale (AIS), a standardized instrument comprising eight items that assess difficulty in initiating and maintaining sleep, overall sleep quality and duration, as well as daytime functioning. The total AIS score ranges from 0 to 24, with higher scores reflecting greater insomnia severity (33–35). A cut-off value of 6 points has been established to facilitate the diagnosis of insomnia with high sensitivity and specificity (36). The AIS has been validated for use in the Polish population (37).

Health-related quality of life was evaluated using the European Quality of Life Five Dimensions—Five Levels (EQ-5D-5L). This standardized instrument comprises five items assessing mobility, self-care, usual activities, pain/discomfort, and anxiety/depression (38). Self-reported health status was assessed using the EuroQol 5-Dimension 5-Level Visual Analog Scale (EQ-VAS), with higher scores reflecting better perceived health (39). The EQ-5D-5L index was calculated according to the algorithm proposed by Golicki et al. (40, 41). The use of the instrument was registered with EuroQol under registration number 70086.

All participants provided informed consent for the treatment protocol and for the pre- and post-treatment evaluations. Bioethical Committee of the Medical University of Silesia in Katowice confirmed that the project did not require ethical approval due to its non-experimental nature (Decision No.: BNW/NWN/0052/KB/41/25, issued on 04 February 2025).

### 2.5 Statistical analysis

Statistical analyses were performed using Statistica 13 PL (StatSoft Polska, Kraków, Poland, 2016) and STATA 19 (StataCorp, College Station, TX, USA, 2023). The normality of continuous variables was assessed with the Shapiro–Wilk test. Depending on the distribution, intra- and intergroup comparisons were conducted using either the Student's *t*-test or the Mann–Whitney *U* test. Categorical variables were compared using the Chi-square (χ^2^) test. Intragroup comparisons for non-parametric data were performed with the Wilcoxon signed-rank test. Changes in the prevalence of insomnia before and after sanatorium treatment were analyzed using the McNemar test. Multivariable logistic regression was used to assess associations between treatment modalities and outcomes, as well as to evaluate the relationship between AIS scores and a history of COVID-19. Statistical significance was defined as *p* < 0.05.

Propensity scores were estimated using logistic regression including age, sex, and BMI as covariates. One-to-one nearest neighbor (greedy) matching without replacement was performed using a caliper width of 0.045 on the logit scale. Balance was assessed using standardized mean differences (SMD) and variance ratios. Before matching, the SMD for the logit of the propensity score was 0.23, indicating moderate imbalance. After matching, the SMD decreased to 0.15, representing a 36% reduction in baseline imbalance. Variance ratios approached unity (1.05), suggesting acceptable dispersion balance. Although a small residual imbalance remained (SMD = 0.15), it was within commonly accepted methodological thresholds (< 0.2), and therefore the matched sample was considered adequately balanced for subsequent analyses.

## 3 Results

**Figure 1:**
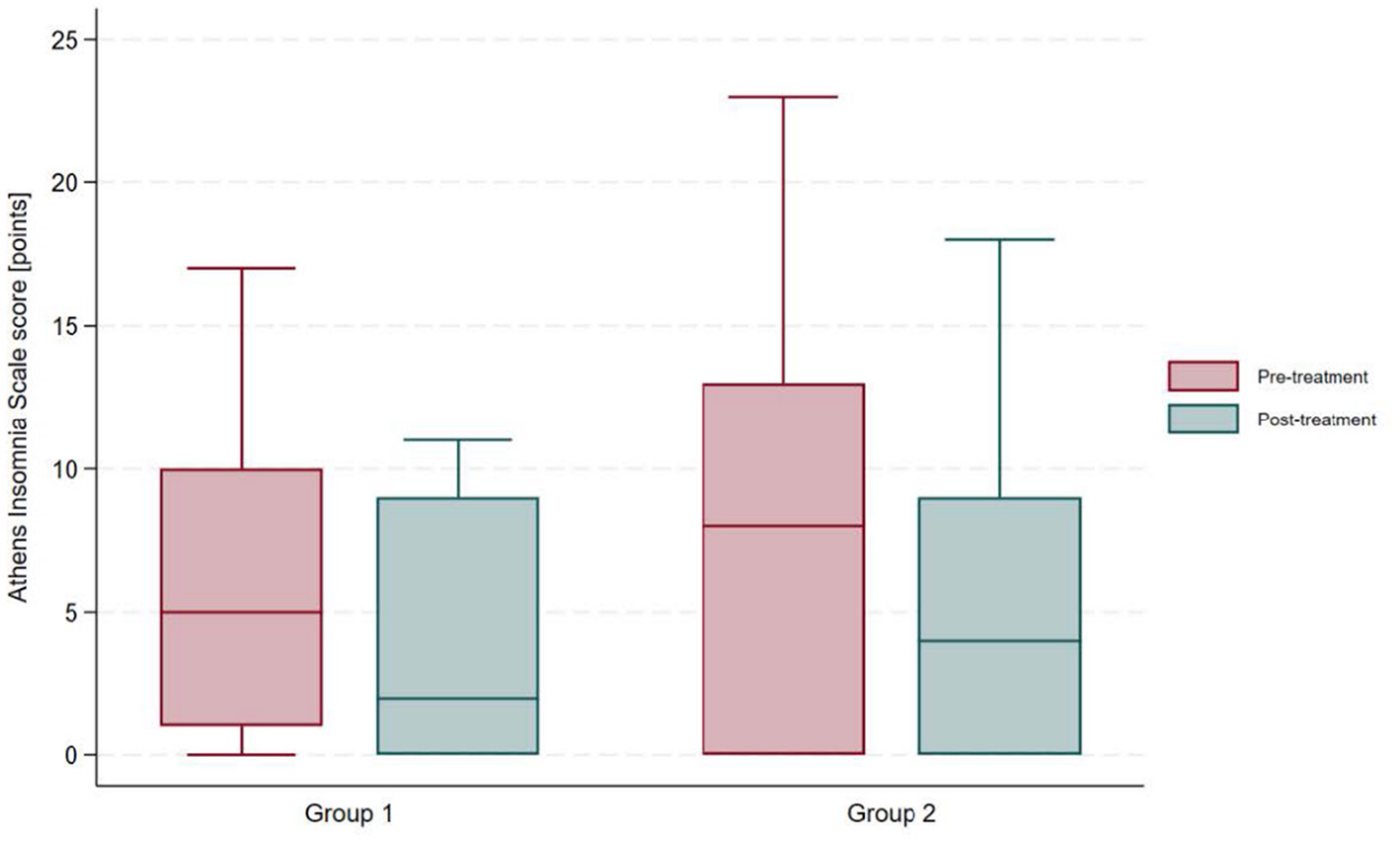
Comparison of Athens Insomnia Scale (AIS) scores before and after treatment in participants with and without a history of COVID-19.

At baseline, participants with a history of COVID-19 had a mean Athens Insomnia Scale (AIS) score of 6.24 ± 5.93. This value was approximately 13% lower than that of participants without a history of COVID-19; the between-group difference was not statistically significant based on an independent samples *t*-test (mean difference: 0.97; 95% CI: −2.42 to 4.35; *t* = 0.58; *p* = 0.57). The prevalence of insomnia at baseline was comparable between groups, with 48% of post-COVID-19 participants and 52% of controls presenting an AIS score ≥6, according to a chi-square test of independence (χ^2^ = 0.07; *p* = 0.80). Comprehensive health resort treatment resulted in a significant improvement in insomnia severity in both groups. Among participants with a history of COVID-19, the AIS score decreased by 36% to a post-treatment mean of 3.97 ± 4.19 points (paired *t*-test: mean change 2.28 ± 3.09; 95% CI: 1.10 to 3.45; *t* = 3.96; *p* = 0.0005). In the control group, insomnia severity decreased by 28%, with a post-treatment mean AIS score of 5.14 ± 5.25 points (paired *t*-test: mean change 2.07 ± 3.33; 95% CI: 0.80 to 3.34; *t* = 3.34; *p* = 0.002). Following treatment, there was no significant difference in AIS scores between the groups based on an independent samples *t*-test (mean difference = 1.17; 95% CI: −1.33 to 3.67; *t* = 0.94; *p* = 0.35). Figure 1 illustrates the AIS results in both study groups.

Following sanatorium treatment, the prevalence of insomnia decreased by 10 percentage points among participants with a history of COVID-19, from 48% (*n* = 14) at baseline to 38% (*n* = 11) post-treatment. However, McNemar's test indicated that this reduction was not statistically significant (χ^2^ = 3.0; exact *p* = 0.25). Among those with baseline insomnia in the post-COVID-19 group, 3 of 14 (21.4%) achieved remission (AIS < 6) post-treatment. In participants without a history of COVID-19, insomnia prevalence declined from 52% (*n* = 15) pre-treatment to 45% (*n* = 13) post-treatment (χ^2^ = 2.0; exact *p* = 0.50); with 2 of 15 (13.3%) participants with baseline insomnia achieved remission. Although post-treatment insomnia prevalence was numerically lower in the post-COVID-19 group, no statistically significant between-group difference was observed at the post-treatment assessment based on a chi-square test of independence (χ^2^ = 0.28; *p* = 0.60).

**Figure 2:**
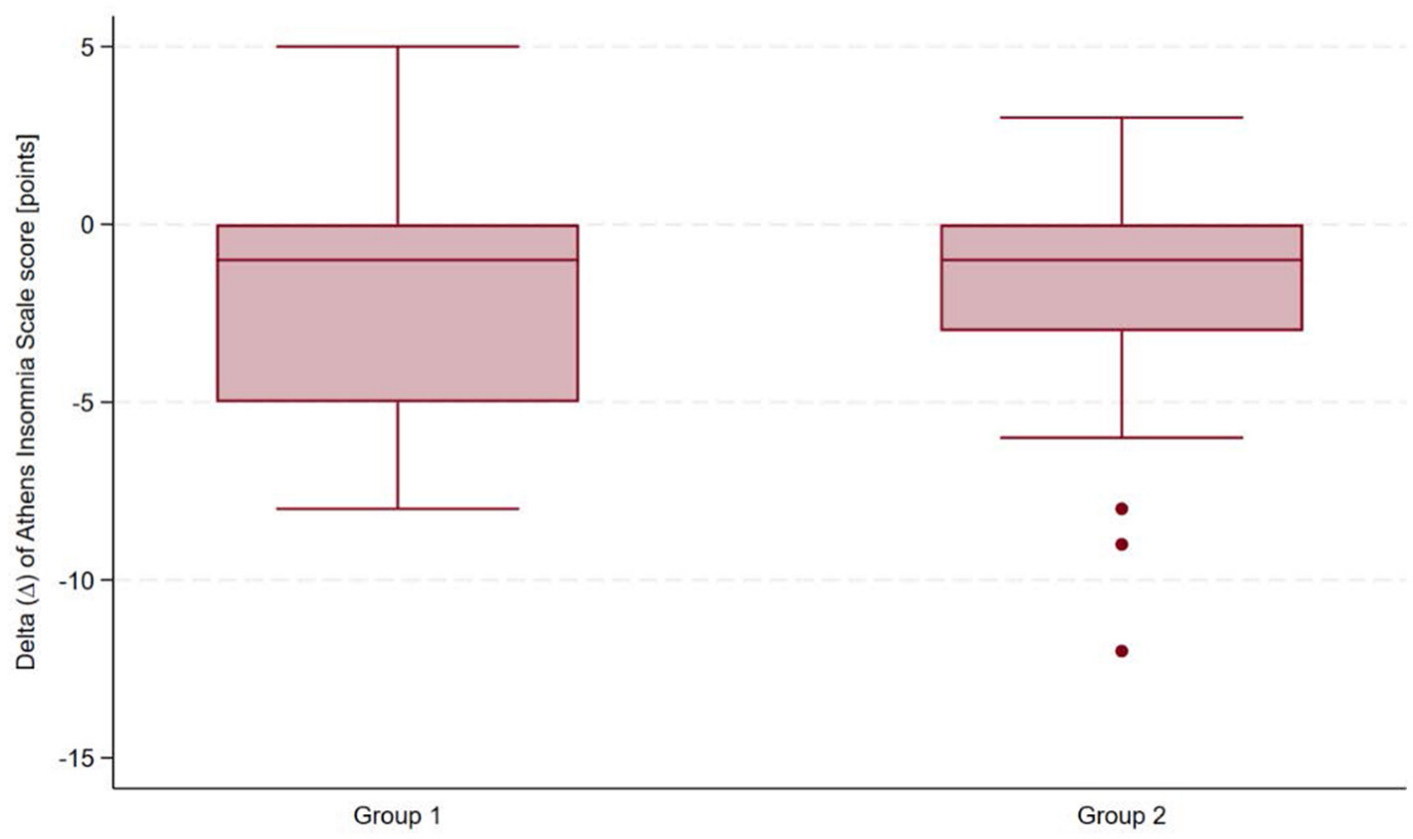
Comparison of the magnitude of change in Athens Insomnia Scale (ΔAIS) scores between participants with and without prior COVID-19 infection.

The magnitude of change in the Athens Insomnia Scale (ΔAIS) was calculated as the difference between post- and pre-treatment measurements to quantify treatment efficacy. Among participants with a history of COVID-19, the mean ΔAIS was approximately 9% lower than in control participants; however, the difference was not statistically significant based on an independent samples *t*-test (mean difference = 0.21; 95% CI: −1.49 to 1.90; *t* = 0.25; *p* = 0.81). Logistic regression analysis indicated that a history of COVID-19 was not significantly associated with the presence of insomnia after sanatorium treatment (OR = 0.75; 95% CI: 0.26–2.15; *p* = 0.59). Figure 2 presents ΔAIS in both groups of participants.

**Figure 3:**
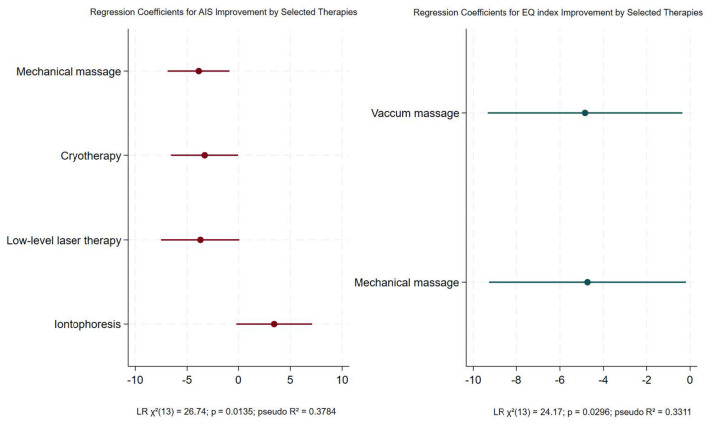
Results of multivariate logistic regression analysis showing regression coefficients and 95% confidence intervals for selected therapies predicting AIS and EQ index improvement.

Multivariable logistic regression analysis revealed that, among the treatment modalities applied during the sanatorium stay, mechanical massage (OR = 0.021; 95% CI: 0.001–0.42; *p* = 0.012) and cryotherapy (OR = 0.037; 95% CI: 0.0014–0.96; *p* = 0.047) were significantly associated with reduced odds of insomnia (AIS ≥ 6), while other modalities were not statistically significant. Low-level laser therapy showed a trend toward reduced odds of insomnia (OR = 0.024; 95% CI: 0.0005–1.09; *p* = 0.055), and iontophoresis demonstrated a non-significant trend (OR = 31.3; 95% CI: 0.81–1200; *p* = 0.064). Improvement in EQ index was significantly associated with mechanical massage (OR = 0.009; 95% CI: 0.00009–0.83; *p* = 0.040) and vacuum massage (OR = 0.008; 95% CI: 0.00009–0.70; *p* = 0.034). A history of COVID-19 and other treatment modalities were not associated with improvements in AIS or the EQ index. Figure 3 presents a forest plot of the logistic regression coefficients for AIS and EQ index improvement by selected therapies. No significant association was found between the applied modalities and the odds of improvement in EQ VAS score (LR χ^2^_(10)_ = 5.24; *p* = 0.8743; pseudo *R*^2^ = 0.1722).

Comprehensive health resort treatment led to a statistically significant improvement in pain and discomfort in both groups of participants. No significant changes were observed in the in the other EQ-5D-5L dimensions. No statistically significant differences between groups were observed at either pre- or post-treatment assessments. Table 5 shows the distribution of responses across the EQ-5D-5L dimensions.

**Table 5:** EQ-5D-5L dimension responses before and after treatment by patient group.

Category	Mobility		Self-care		Usual activities		Pain/discomfort		Anxiety/depression	
	**Before**	**After**	**Before**	**After**	**Before**	**After**	**Before**	**After**	**Before**	**After**
Group 1										
No problems	68%	72%	92%	88%	88%	84%	32%	48%	96%	100%
Slight problems	16%	16%	8%	12%	12%	16%	44%	48%	4%	0%
Moderate problems	12%	8%	0%	0%	0%	0%	20%	4%	0%	0%
Severe problems	4%	4%	0%	0%	0%	0%	4%	0%	0%	0%
Extreme problems/unable to do	0%	0%	0%	0%	0%	0%	0%	0%	0%	0%
*z*	1.41		−1.00		−1.00		2.93		1.00	
*p* Value^a^	0.50		1.00		1.00		0.004		1.00	
Group 2										
No problems	81%	89%	89%	93%	96%	96%	59%	78%	93%	93%
Slight problems	15%	7%	11%	7%	4%	4%	30%	22%	7%	7%
Moderate problems	4%	4%	0%	0%	0%	0%	11%	0%	0%	0%
Severe problems	0%	0%	0%	0%	0%	0%	0%	0%	0%	0%
Extreme problems/unable to do	0%	0%	0%	0%	0%	0%	0%	0%	0%	0%
*z*	1.41		1.00		0.00		2.83		0.00	
*p* value^a^	0.50		1.00		1.00		0.008		1.00	
Group 1 vs. Group 2										
*z*	−1.26	−1.56	0.38	−0.56	−1.11	−1.49	−2.07	−2.26	0.52	1.37
*p* value^b^	0.22	0.16	1.00	0.58	0.55	0.30	0.05	0.04	1.00	0.53

^a^p value—intragroup comparisons before vs. after treatment; analyzed using the Wilcoxon signed-rank test for paired data.

^b^p value—intergroup comparisons between Group 1 and Group 2; analyzed using the Wilcoxon rank-sum test (Mann–Whitney U test) for independent data.

**Figure 4:**
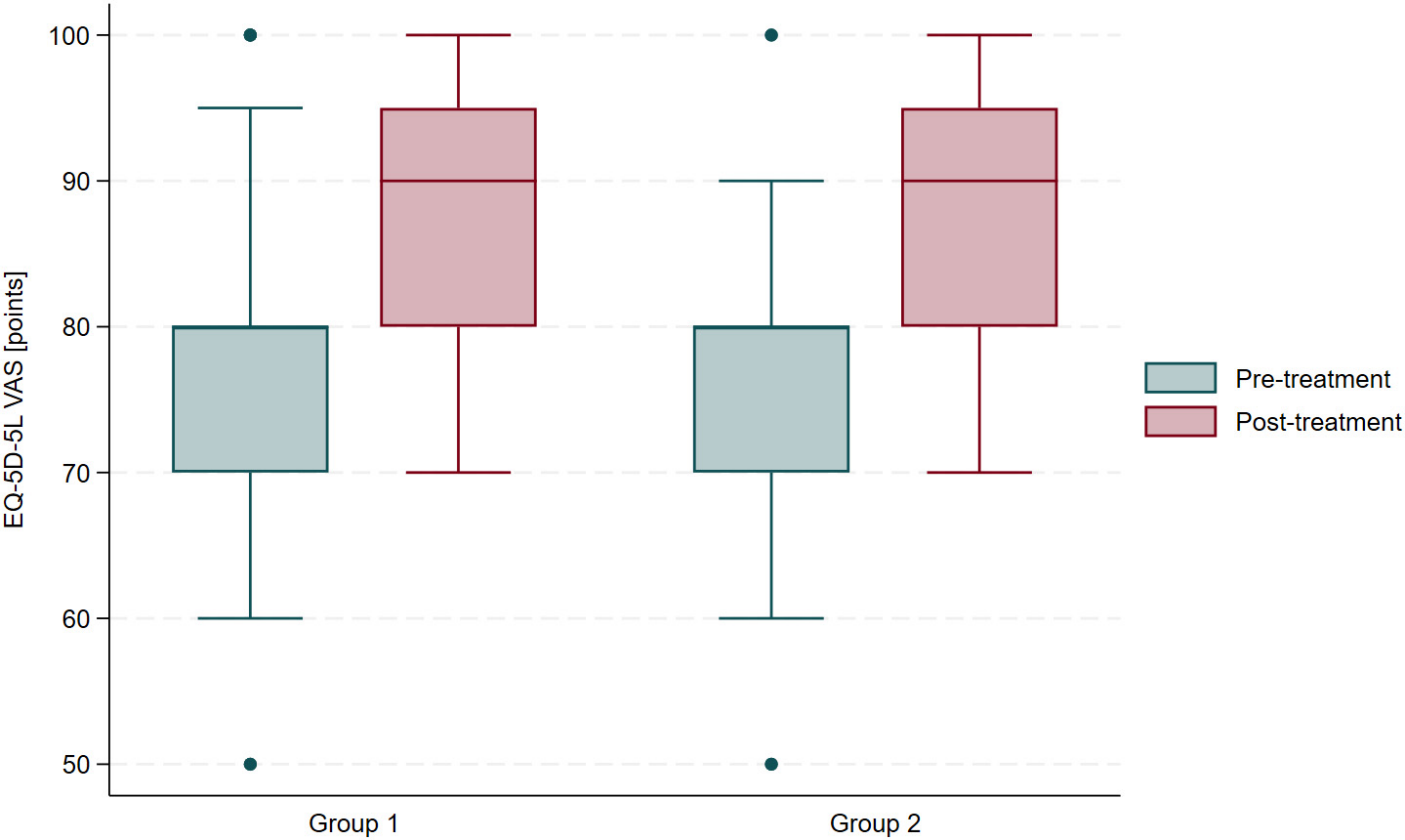
Comparison of EQ VAS scores before and after treatment in participants with and without a history of COVID-19.

At the pre-treatment evaluation, an independent samples *t*-test showed no significant differences in VAS scores between the groups of participants (mean difference: 0.35; 95% CI: −5.69 to 6.38; *t* = 0.11; *p* = 0.91). Comprehensive health resort treatment led to improvement in VAS scores in individuals with a history of COVID-19 (paired *t*-test; mean change: −12.41 ± 7.63; 95% CI: −15.32 to −9.51; *t* = −8.76; *p* < 0.0001) and in the control group (paired *t*-test; mean change: −12.41 ± 6.76; 95% CI: −14.99 to −9.84; *t* = −9.88; *p* < 0.0001). Following the sanatorium stay, VAS scores did not differ between groups based on an independent samples *t*-test (mean difference: 0.35; 95% CI: −3.75 to 4.44; *t* = 0.17; *p* = 0.87). No significant differences were observed between groups with respect to ΔVAS (independent samples *t*-test; mean difference: 0; 95% CI: −3.79 to 3.79; *t* = 0.00; *p* = 1.00). Figure 4 presents EQ VAS scores before and after treatment, stratified by group.

**Figure 5:**
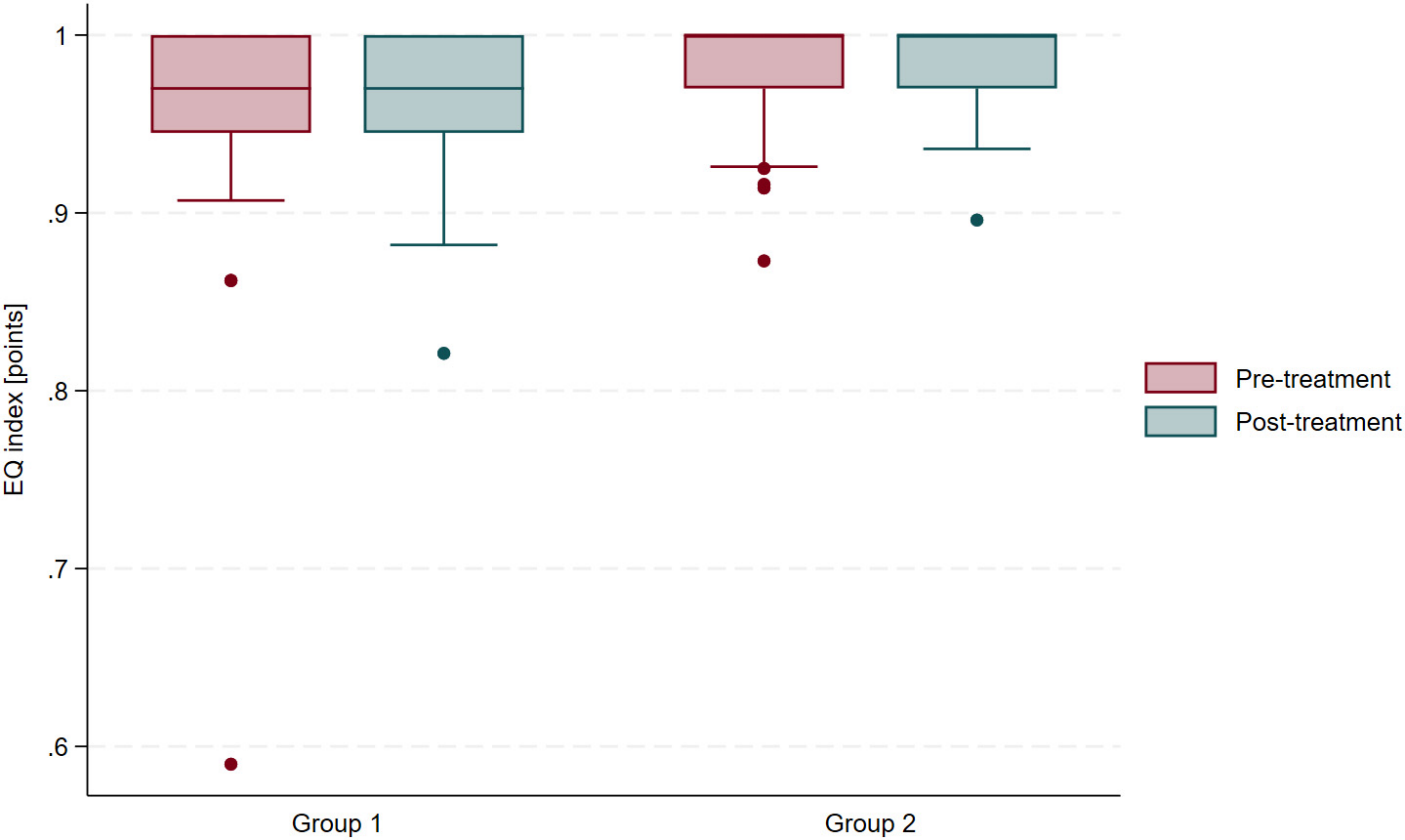
Changes in EQ index scores before and after treatment in participants with and without a history of COVID-19.

During the pre-treatment evaluation, individuals with a history of COVID-19 had an approximately 2% lower EQ index than the control group; however, this difference was not statistically significant based on independent samples *t*-test (mean difference = 0.03; 95% CI: −0.008 to 0.064; *t* = 1.56; *p* = 0.13). Comprehensive health resort treatment resulted in improvements in EQ index in both groups. In participants with a history of COVID-19 (Group 1), the improvement was approximately 2% (paired *t*-test; mean change = −0.02 ± 0.05; 95% CI: −0.036–0.002; *t* = −1.85; *p* = 0.08), while in the control group (Group 2), it was approximately 1% (paired *t*-test; mean change = −0.01; 95% CI: −0.022 to −0.00001; *t* = −2.06; *p* = 0.05). Post-treatment comparison showed that COVID-19 survivors had slightly lower EQ index than the control group (independent samples *t*-test; mean difference = 0.021; 95% CI: 0.0005–0.042; *t* = 2.06; *p* = 0.05). The change in EQ index (ΔEQ) did not differ significantly between the groups based on an independent samples *t*-test (mean difference = −0.006; 95% CI: −0.027 to 0.015; *t* = −0.61; *p* = 0.55). Figure 5 presents EQ index scores before and after treatment, stratified by group.

## 4 Discussion

The findings of this study suggest that health resort treatment may contribute to improvements in insomnia symptoms and perceived health status among COVID-19 survivors, while exerting a limited effect on overall quality of life. Notably, participation in a 21-day comprehensive sanatorium-based program was associated with comparable improvements in insomnia and quality of life across both groups, indicating that the observed benefits may be attributable to the general rehabilitative environment and multimodal interventions rather than to group-specific factors.

Previous reports indicate an increased prevalence of sleep disorders among individuals with long COVID syndrome (1, 17, 26, 35, 42). At baseline, neither insomnia severity nor prevalence differed significantly between individuals with a history of COVID-19 and the control group. The prevalence of insomnia in post-COVID participants was 48%, higher than the 39.2% reported by Kalamara et al. (43) 6 months after hospital discharge. This discrepancy may be explained by differences in age: the mean age in the present study was 68.62 ± 10.32 years, while in the study by Kalamara et al. it was 56.0 ± 11.48 years. As age is a well-established determinant of sleep disorders (44), which may account for the higher prevalence observed in the present study. Baseline insomnia severity was relatively low and highly variable in both groups, potentially introducing a floor effect that could limit the interpretation of observed changes. Despite these limitations, both groups showed significant improvements in AIS scores following the intervention, suggesting a meaningful treatment effect. Future studies with larger samples and higher baseline insomnia severity are warranted to confirm these findings and evaluate longer-term outcomes.

Comprehensive health resort treatment was associated with an improvement in insomnia symptoms in both individuals with a history of COVID-19 and control participants; however, no statistically significant differences were observed between the groups. Several factors may explain this finding. First, sanatoria are legally required to be located in designated areas that comply with statutory regulations established under the Act of 28 July 2005 on Health Resort Treatment, Health Resorts and Health Resort Protection Areas, and Health Resort Municipalities. In accordance with the Act, sanatoria must be situated within Zone “A,” which is subject to stringent regulatory criteria, including the mandatory observance of night-time quiet hours from 10:00 p.m. to 6:00 a.m. (45). Irregular sleep schedules have been associated with a broad spectrum of adverse health outcomes, including cardiovascular disease (46), obesity and type 2 diabetes (47), as well as mental health disorders (48). Accordingly, the implementation of night-time quiet hours in sanatoria may constitute a contributing factor to the observed improvement in sleep outcomes, as regularity of sleep–wake patterns represents a fundamental component of sleep hygiene and has been shown to enhance both sleep duration and sleep quality (49, 50). Furthermore, municipalities with health resort status must comply with environmental noise standards (45), and exposure to environmental noise has been demonstrated to be associated with impaired sleep quality (51, 52). Health resorts are also required to meet criteria concerning protection against electromagnetic fields (45), which have been reported to exert detrimental effects on sleep parameters (53–55). In addition, Zone “A” areas are required to include substantial green spaces, and exposure to green environments has been shown to exert beneficial effects on sleep (56). Taken together, adherence to these stringent statutory requirements may play an adjuvant role in the improvement of sleep outcomes, as the location of sanatoria ensures the provision of treatment in environments conducive to health promotion, thereby offering a plausible explanation for the observed results.

During their stay in a sanatorium, post-COVID-19 participants received various therapeutic modalities, with mud therapy and brine treatments being the most frequently applied. Numerous studies have demonstrated that balneological factors improve sleep quality (57–62). Castelli et al. (57) suggested that sleep improvement from balneotherapy results from thermoregulatory mechanisms, as body temperature is regulated by the circadian rhythm. Given that the permeability of the blood–brain barrier to cytokines is also circadian-dependent (63), therapies influencing thermoregulation may represent an effective adjuvant approach in the management of sleep disorders. Ditmer et al. (64) indicated that interleukin-6 (IL-6) plays a significant role in sleep disturbances, while tumor necrosis factor-α (TNF-α) may also contribute to their pathogenesis (63). Balneological treatments have consistently been shown to reduce levels of pro-inflammatory cytokines and, consequently, may directly contribute to improvements in sleep quality (26, 65). In addition, balneotherapy modulates the hypothalamic–pituitary–adrenal axis. Cortisol, which exerts anti-inflammatory and anti-edematous effects (66), physiologically follows a circadian pattern with higher morning levels and a gradual decline in the evening (67). Importantly, elevated morning cortisol levels have been associated with severe insomnia (68). Antonelli and Donelli (69) demonstrated that balneotherapy favorably influences cortisol regulation.

Despite these potential mechanisms, the present study did not demonstrate a significant association between treatments based on natural healing resources (brine and peloids) and reductions in insomnia, consistent with regression analysis. In contrast, mechanical massage and cryotherapy were significantly associated with reduced odds of insomnia. Van Dijk et al. (70) reported modest effects of mechanical massage on cortisol levels, which may partly explain its beneficial effects on sleep. Van Pelt et al. (71) showed that massage therapy improves inflammatory cytokine profiles, including TNF-α and IL-6, potentially contributing to better sleep (63, 64). Whole-body cryotherapy has been previously shown to improve insomnia through pain reduction and modulation of parasympathetic activity (72). In this study, cryotherapy was applied locally, so observed effects cannot be directly compared with systemic effects; however, local cryotherapy may reduce inflammation and indirectly improve sleep (63, 64, 73).

All participants also engaged in supervised exercise and leisure walking. Physical activity is well-documented to benefit sleep (74, 75). Considering the multifactorial pathophysiology of post-COVID syndrome, including blood–brain barrier disruption, coagulopathy, vascular dysfunction, persistent inflammation, immune dysregulation, and autonomic imbalance (1, 2, 26, 63), the observed improvements in sleep following sanatorium-based treatment appear biologically plausible and clinically relevant. Improvements in insomnia were comparable between groups, supporting the use of comprehensive health resort therapy in post-COVID participants, as it is not associated with serious adverse events (76). Given the high sensitivity and specificity of the AIS for diagnosing insomnia (29, 36), future studies should incorporate objective sleep measures, such as actigraphy or polysomnography, to strengthen validity.

Stay in a sanatorium did not significantly affect overall quality of life in post-COVID-19 participants, as the EQ index remained unchanged. A trend toward improvement was observed in the control group but did not reach statistical significance. The magnitude of change in EQ-5D-5L index was similar between groups. These findings contrast with previous studies showing quality-of-life improvements after spa therapy and balneotherapy (69, 77, 78). However, the relatively high baseline values observed in the participants of the present study may have limited the potential for further improvement, which could partially explain the discrepancies compared to findings reported in other studies. During the sanatorium treatment, a multimodal therapeutic approach was applied. However, improvement in the EQ index was significantly associated only with vacuum and mechanical massages. Rodríguez-Huguet et al. (79) reported that negative pulsed-pressure myofascial vacuum therapy may positively affect quality of life. To date, no studies have evaluated the impact of mechanical massage on quality of life. Given the known physiological effects of massage, the observed improvement in quality of life may be mediated by these mechanisms (80). These results should be interpreted cautiously due to the small sample size and wide confidence intervals.

Comprehensive health resort treatment resulted in a significant reductions in pain intensity and an improvements in self-assessed health status measured by VAS scale. Therapeutic modalities applied during the sanatorium stay have been shown to reduce pain through multiple mechanisms (26, 65, 81–83). Heat application influences the hypothalamic-pituitary-adrenal axis, and the subsequent release of β-endorphins may contribute to analgesic effects (84). Additionally, heat-based therapies induce vasodilation, leading to improved tissue oxygenation, which may further facilitate pain reduction (85). Analgesic effects may also result from the inhibition of nociceptive transmission via large-diameter afferent fibers following the application of therapeutic agents (86). Furthermore, supervised exercise may contribute to pain alleviation through its effects on the central nervous system, endocrine regulation, and modulation of inflammatory markers (87). Pain reduction in post-COVID participants likely reflects combined effects of these interventions. Future studies should incorporate additional measures, such as numeric rating scales or objective pain thresholds, to enhance reliability.

Although the EQ index did not improve in post-COVID-19 participants, overall health status assessed using the visual analog scale (VAS) increased. Pain is closely associated with perceived health status (88, 89), suggesting that the observed VAS improvements may be primarily driven by pain alleviation. This is further supported by the stability of the remaining EQ-5D-5L domains, which likely reflects the eligibility criteria for health resort treatment, requiring patients to maintain independent mobility and self-care (90). While participants engaged in supervised exercise sessions, their impact on mobility was minimal, as this domain did not change significantly. Importantly, the change in VAS (ΔVAS) did not differ between individuals with a history of COVID-19 and control participants.

Treatment in the sanatorium lasted 21 days. Previous studies suggest that comprehensive health resort therapy can be effective in post-COVID-19 individuals even with short-term interventions (91, 92), though most reports focus on longer treatment durations (30, 82, 93). Onik and Sieroń (94) reported that, in patients with long COVID, extended treatment was associated with greater improvements in outcomes. Most studies in post-COVID-19 individuals have focused on the immediate effects of interventions, although Ovejero et al. (30) included a 2-month follow-up in patients undergoing balneotherapy. While the primary aim of the present study was to evaluate the immediate effects of comprehensive sanatorium-based treatment, future research should incorporate at least a 1-month follow-up to assess the persistence of treatment effects and strengthen the reliability of the findings.

The present study has several limitations. First, the relatively small sample size and strict exclusion criteria may limit the generalizability of the findings. The modest sample size also reduced statistical power, and although some differences reached statistical significance, the results should be interpreted with caution. Future studies involving larger and more diverse populations are warranted to confirm these findings and enhance their generalizability. Second, the absence of a non-treated control group limits the ability to infer causal effects. Future research should include a non-treatment control group and be conducted as a randomized controlled trial to strengthen the validity of the findings. Third, this study may be subject to selection bias due to the rules governing patient referrals to sanatorium treatment. Exclusion criteria were applied to remove conditions that could influence the clinical course of long COVID, sleep, or quality of life; however, they may also contribute to selection bias. Although it cannot be completely ruled out, this potential bias should be considered when interpreting the results. Fourth, multiple comparisons represent a potential limitation, as they may increase the risk of type I error. Although the analyses addressed conceptually distinct hypotheses, the results should be interpreted with caution. Fifth, although some individual modalities were associated with outcomes, the multimodal nature of the intervention should be considered the primary driver of the observed effects. Sixth, since age is a known confounder in sleep disorders, future studies should include younger participants to more precisely evaluate the impact of health resort-based interventions on sleep outcomes. Seventh, the relatively high baseline EQ-5D-5L scores suggest a potential ceiling effect, which may have limited the ability to detect improvements in quality of life following the intervention. Conversely, the relatively low baseline AIS scores may have introduced a floor effect, reducing the sensitivity to detect changes in insomnia severity.

## 5 Conclusions

Comprehensive health resort treatments appear to offer benefits in alleviating insomnia and improving self-reported health status among COVID-19 survivors. While sanatorium-based comprehensive curation does not seem to lead to substantial improvements in overall quality of life for individuals with a history of COVID-19, the outcomes observed are broadly comparable to those seen in individuals without prior COVID-19 infection. These findings suggest potential value in this therapeutic approach, although further research would be helpful to better understand its efficacy and clinical utility in post-COVID-19 populations.

## Data Availability

The raw data supporting the conclusions of this article will be made available by the authors, without undue reservation.
